# A systematic review of endpoint definitions in late phase pulmonary tuberculosis therapeutic trials

**DOI:** 10.1186/s13063-021-05388-1

**Published:** 2021-08-03

**Authors:** N. K. Hills, J. Lyimo, P. Nahid, R. M. Savic, C. Lienhardt, P. P. J. Phillips

**Affiliations:** 1 UCSF Department of Epidemiology & Biostatistics, San Francisco, California USA; 2 MDR-TB Coordinator-National TB and Leprosy Program, Ministry of Health, Dodoma, Tanzania; 3 UCSF Center for Tuberculosis, University of California San Francisco, San Francisco, California USA; 4 Unité Mixte Internationale TransVIHMI (UMI 233 IRD – U1175 INSERM - Université de Montpellier), Montpellier, France

## Abstract

**Background:**

Safe, more efficacious treatments are needed to address the considerable morbidity and mortality associated with pulmonary tuberculosis (TB). However, the current practice in TB therapeutics trials is to use composite binary outcomes, which in the absence of standardization may inflate false positive and negative errors in evaluating regimens. The lack of standardization of outcomes is a barrier to the identification of highly efficacious regimens and the introduction of innovative methodologies

**Methods:**

We conducted a systematic review of trials designed to advance new pulmonary TB drugs or regimens for regulatory approval and inform practice guidelines. Trials were primarily identified from the WHO International Clinical Trial Registry Platform (ICTRP). Only trials that collected post-treatment follow-up data and enrolled at least 100 patients were included. Protocols and Statistical Analysis Plans (SAP) for eligible trials from 1995 to the present were obtained from trial investigators. Details of outcome data, both explicit and implied, were abstracted and organized into three broad categories: favorable, unfavorable, and not assessable. Within these categories, individual trial definitions were recorded and collated, and areas of broad consensus and disagreement were identified and described.

**Results:**

From 2205 trials in any way related to TB, 51 were selected for protocol and SAP review, from which 31 were both eligible and had accessible documentation. Within the three designated categories, we found broad consensus in the definitions of favorable and unfavorable outcomes, although specific details were not always provided, and when explicitly addressed, were heterogeneous. Favorable outcomes were handled the most consistently but were widely variable with respect to specification. In some cases, the same events were defined differently by different protocols, particularly in distinguishing unfavorable from not assessable events. Death was often interpreted as conditional on cause. Patients who did not complete the study because of withdrawal or loss to follow-up presented a particular challenge to consistent interpretation and analytic treatment of outcomes.

**Conclusions:**

In a review of 31 clinical trials, we found that outcome definitions were heterogeneous, highlighting the need to establish clearer specification and a move towards universal standardization of outcomes across pulmonary TB trials. The ICH E9 (R1) addendum provides guidelines for undertaking and achieving this goal.

**PROSPERO registration:**

PROSPERO CRD42020197993. Registration 11 August 2020.

**Supplementary Information:**

The online version contains supplementary material available at 10.1186/s13063-021-05388-1.

## Introduction

Tuberculosis (TB) kills more people globally than any other single pathogen [1], with mortality and morbidity likely to increase as a result of the COVID-19 pandemic and the many ensuing challenges posed to the national TB control programs [2]. New shorter, safer, and more efficacious treatments are urgently needed [3]. In response to this need, more than a dozen new compounds are in early or middle clinical development (https://www.newtbdrugs.org/pipeline/clinical) with numerous late-phase randomized controlled trials expected in the near future, conducted either by individual pharmaceutical companies, or as part of publicly or philanthropically funded networks.

Most recent and ongoing late-phase TB therapeutics trials have used a composite binary outcome that usually combines bacteriological failure and relapse, death, and treatment changes as “unfavorable” in the primary efficacy outcome. While seemingly binary (unfavorable vs. favorable), there is commonly a third category of “not assessable” which includes losses to follow-up and other outcomes that result in missing data and results in the exclusion of patients with this outcome from some analyses. Additionally, multiple analysis populations are usually proposed as co-primary. These include an intention-to-treat analysis (ITT) population including all patients randomized, classifying as unfavorable any participants with substantial missing data; a modified ITT (mITT) analysis population excluding some losses to follow-up from the analysis, and a per protocol (PP) analysis population excluding participants who had a protocol violation or did not complete a sufficient proportion of treatment. This approach has a number of limitations:

- Not standardized. Outcome definitions are not standardized across phase III TB treatment trials. This leads to considerable challenges in combining data, interpreting results, assessing comparative efficacy, implementing predictive modeling, and conducting necessary meta-analyses (as exemplified in the TB-ReFLECT project [4]).
- Outdated. The emphasis on simple, unadjusted per protocol analyses (not considering causal inference methods [5]) and even modified intention-to-treat analyses with post-randomization exclusions is at odds with best practice in other disease areas [5, 6] and regulatory guidance [7]. The draft version of the FDA guidance document for non-inferiority trials (2010) initially accommodated an “as-treated” analysis, but this was removed in the final guidance document (2016) [7].
- May inflate type I and II errors. Classifying the outcome of participants lost to follow-up as unfavorable (i.e., defining “missing” as “failure”) is likely to result in conservative estimates in superiority trials by diluting any treatment effect (and is therefore often favored by regulators). This is not necessarily conservative in a non-inferiority trial, can inflate type I and type II errors, and also results in misleading decisions in the context of adaptive platform trial designs.
- A barrier to identifying highly efficacious regimens. Including events that are less likely to be related to treatment (including the loss to follow-up and non-TB mortality) in a composite outcome increases variability in treatment effect estimates and therefore necessitates an increased sample size. This added “noise” also makes it challenging to identify interventions (like stratified medicine approaches [4]) that may result in very high cure rates (97–100%) without requiring prohibitively large sample sizes [8].
- At odds with policy makers and guideline developers. WHO guidelines generally rely on WHO programmatic outcomes definitions [9] when considering evidence (the 2018 DR-TB guidelines is a case in point [10, 11]). The “catch-all” nature of the composite outcome currently used in phase III trials is likely to have contributed to this disconnect between trials and the approach taken for guidelines.
- Mixes efficacy and safety events. Including treatment changes due to adverse events during treatment in the composite outcome conflates safety and tolerability with efficacy.
- Impedes progress in prediction modeling. A phase III outcome defined by composite events does not allow for efficient and predictive linkage with phase IIB endpoints, such as time to culture conversion, that are essential for bridging the gap between phase II and phase III trials [12], and that will be increasingly important as new biomarkers of TB treatment response are identified [13]. Similarly, translational modeling across the species (NHP, mice, rabbit) is limited due to discordance in outcomes, enabling translational errors and suboptimal decision-making regarding which regimens to advance in clinical development.

Furthermore, regulatory guidance is changing with the ICH E9 (R1) addendum on Estimands and Sensitivity Analyses (finalized November 2019), which formalizes a new approach to specifying trial objectives, endpoints, and analysis populations [14]. The estimand (as it is named in the Addendum) defines in detail what will be estimated in order to address a specific scientific question; it is constructed based on what is of clinical relevance for the treatment of interest in the trial. An estimand comprises five attributes: treatment, population, an accounting for events which affect or preclude measurement of the outcome (“intercurrent events”) and specification of the population-level summary which will provide a basis for comparison between treatment conditions. As such, it provides a language that can be used to refine the specification of the phase III primary efficacy outcome, an imperative task given the new late-phase trials expected on the horizon.

The objective of this systematic review was to first catalog long-term definitive outcome definitions (including analysis populations and primary objectives) from recent phase IIC and III trials for new regimens for drug-susceptible (DS) and drug-resistant (DR) tuberculosis and then to conduct a thematic analysis on these outcomes to identify areas of consensus and disagreement. The overarching goal of this work is to use these results to develop standardized consensus estimands for phase IIC and III TB therapeutics trials.

## Methods

The protocol for the systematic review was prospectively registered on the PROSPERO registry (PROSPERO 2020 CRD42020197993) [15] and is provided as an online supplement, along with the PRISMA checklist [16].

Briefly, this systematic review sought to identify trials that have been designed to advance a new drug or regimen for regulatory approval and therefore inform and impact practice guidelines. The focus was on treatment trials for pulmonary TB, including phase II, phase III, and other late-phase randomized controlled trials (RCTs), or non-randomized trials of new drugs intended specifically for regulatory approval. Trials of treatment for latent TB, prevention of TB, diagnosis of TB, extrapulmonary TB, adjuvant nutritional supplements or immune therapies, ART initiation among TB patients, and trials of TB vaccines and programmatic interventions looking at adherence interventions (DOT or mHealth initiatives) were excluded as endpoints in these trials are defined differently. Trials that did not collect outcome data on post-treatment follow-up (for relapse) or that enrolled fewer than 100 patients were excluded since these were clearly not designed to change guidelines and practice.

The WHO International Clinical Trial Registry Platform (ICTRP) was the primary database searched to identify relevant trials. To increase the likelihood that no trials were missed, we also contacted experts in the field of TB trials to identify other trials and reviewed the excellent list of DR-TB clinical trials maintained by RESIST-TB (www.resisttb.org).

Two individuals (PPJP and JJL) independently reviewed the list of trials identified from the search strategy using titles and other fields from the ICTR platform to determine whether they met the inclusion criteria. Investigators or sponsor representatives of final selected studies were contacted to access the study statistical analysis plans (SAPs) and study protocols; these were downloaded from the public domain when available. Two individuals (NKH and JJL) reviewed all protocols and Statistical Analysis Plans and abstracted relevant information.

Qualitative data from primary endpoint definitions of different studies were analyzed using thematic analysis in the five stages outlined by Braun and Clarke [17]. Qualitative analyses and summaries were done by NKH. The final draft of the manuscript was circulated to PIs of all completed trials for their comments, approval, and edits. Our objective was to describe areas of consensus and disagreement as drawn from protocols and SAPs across trials, rather than to critique individual trials. For this reason, we do not discuss nuances in definitions in specific trials, but rather aim to provide a summary of broad trends in outcome definitions and analyses used in recent TB treatment trials.

## Results

**Fig. 1:**
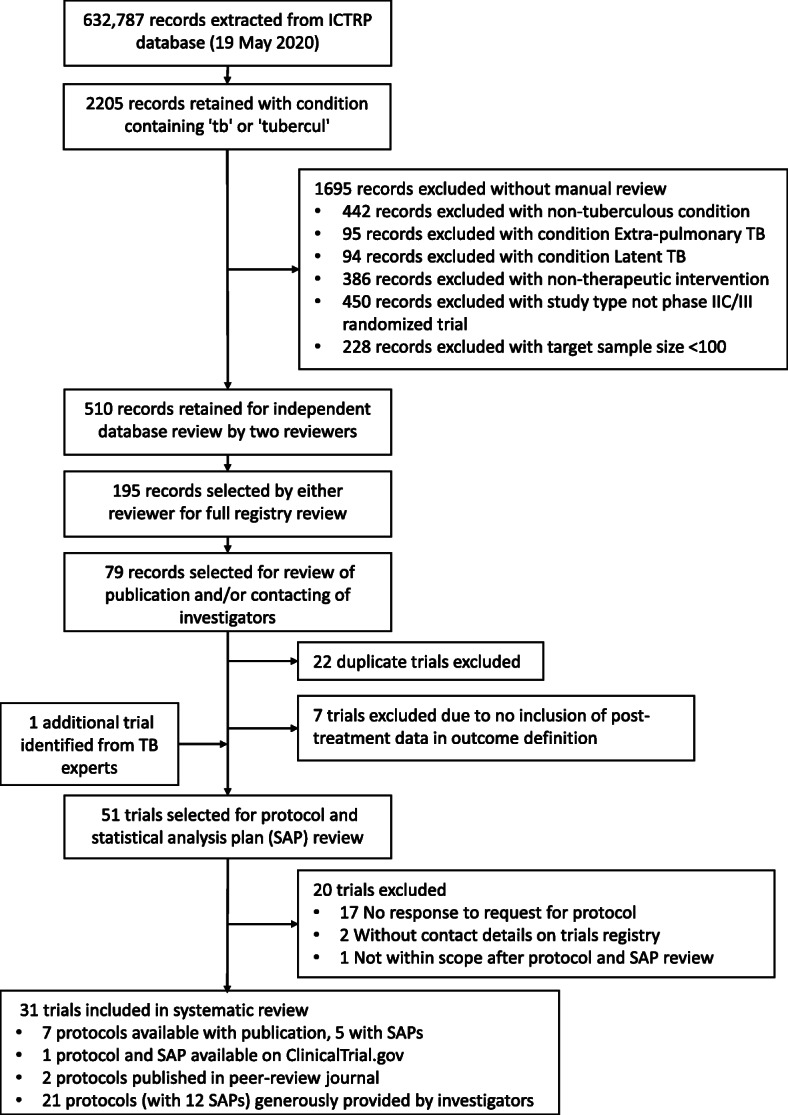
PRISMA flow diagram

Due to heavy traffic generated by the COVID-19 pandemic during early 2020 and limited ability to use the online search portal for the WHO ICTR, we downloaded the full ICTR database (3.5GB, 19 May 2020) and used it for this systematic analysis. From 632,787 clinical trials registered, we identified 2205 with condition containing “tb” or “tubercul” and selected 510 for independent registry review by two reviewers. All registry information was available in English. From these, we identified 51 trials that were highly likely to be relevant and eligible for inclusion (See Fig. 1 for PRISMA flow diagram [16]). We then contacted Principal Investigators of the selected trials to request the most current versions of their protocols and, when possible, SAPs. We received protocols from 31 studies and SAPs from 18 (58%). Many trials were listed on more than one trial registry; the majority (27, 87%) were listed at least on ClinicalTrials.gov; two of the studies were only listed on the International Standard Randomised Controlled Trial Number (ISRCTN) registry (isrctn.com), and two trials were only listed on the Clinical Trials Registry of India (ctri.nic.in). Registration across all trials was finalized between the years of 2001 and 2020 (although in some early cases, trials were not registered until after completion; the earliest trial began enrolling in 1995 but was not registered until 2001), with 21 (68%) of the trials registered during or after 2010. All protocols were available in English. The majority of the trials (26, 84%) were phase III, either with (n=29) or without (n=2) internal controls; one trial was described as phase IIB/III, two were listed as phase IIC, and two as phase IV (see Table 1). Throughout the paper, we will refer to the trials as a group as “phase III,” while recognizing that several phase II and phase IV trials have been included. Only one trial was pharmaceutically sponsored.

**Table 1:** List of included trials and trial characteristics

Trial registry ID^a^	Trial dates^b^	Location of sites	Sample size	Participant age (years)	Inclusion of participants with HIV infection	Intervention	Control	Total trial duration	Analysis	Current status
***DS-TB***										
**TBTC Study 22, NCT00023335**	Reg: 10Sept2001 [reg. after end of trial] SC: March 2001	USA Canada	1004	≥ 18	No, excluded	Once-weekly rifapentine and isoniazid in CP	Standard 6-month control (twice-weekly CP)	30 months	Non-inferior [6.6%]	Completed and published [18]
**ACTG222, NCT00001033**	Reg: 31Aug2001 SC: July1997	USA	650	≥ 13	Yes, with restrictions	A 9-month intermittent regimen with or without addition of levofloxacin	Standard 6-month control	1.5 years	Equivalence [<10%]	Completed and published [19]
**Study C, NCT00216333**	Reg: 22Sep2005 PC: 6Sep2008 SC: 6Sep2009	Africa, Asia, Latin America	1585	18–65	Yes, if CD4 ≥350	Fixed dose combination 6-month regimens	Standard 6-month control (separate tablets)	30 months	Non-interior [4%]	Completed and published [20]
**Oflotub, NCT00216385**	Reg: 22Sep2005 SC: Dec2008	Benin, Kenya, South Africa,Senegal Guinee-Conakry	2070	18–65	Yes, with restrictions	A 4-month gatifloxacin regimen	Standard 6-month control	2 years	Non-inferior [6%]	Completed and published [21]
**DMID-01-009, NCT00130247**	Reg: 15Aug2005 PC: 2Sep2008 SC: 28Nov2008	Uganda Brazil Philippines	394	18–60	No, excluded	A 4-month standard 4-drug regimen	Standard 6-month control	30 months	Equivalence [5%]	Completed and published [22]
**NIRT TRC Study 24, CTRI/2008/091/000024**	Reg: 5Sep2008	India	1371	18–60	No, excluded	A 4-month regimens with moxifloxacin	6-month regimen: 2 RHZE thrice weekly/4 RH thrice weekly	28 months	Equivalence [5%]	Completed and published [23]
**Rifaquin, ISRCTN44153044**	Reg: 15Aug2008 PC: 1Nov2012 SC: 1Nov2012	South Africa, Zimbabwe, Botswana, Zambia	827	≥ 18	Yes, if CD4 ≥200	A 4-month and 6-month intermittent regimens with rifapentine and moxifloxacin	Standard 6-month control	18 months	Non-inferior [6%]	Completed and published [24]
**REMOX, NCT00864383**	Reg: 18Mar2009 PC: Oct2013 SC: Feb2014	China, India, Malaysia, Mexico, Thailand, Zambia, Tanzania, Kenya, South Africa	1931	≥ 18	Yes, if CD4 ≥250	A 4-month regimens with moxifloxacin	Standard 6-month control	18 months	Non-inferior [6%]	Completed and published [25]
**NIRT Study 25, NCT00933790**	Reg: 7Jul2009 PC: 31Dec2016 SC: 30June2018	India	331	≥ 18	Yes	Daily, partly daily and intermittent 6-month regimens	Standard 6-month control	18 months	Superiority using survival analysis	Completed and published [26]
**Study A, ISRCTN19832141**	Reg:15Apr2010 SC: 1Dec2001	Benin, China, Guinea, Mozambique, Nepal, Tanzania	1355	15–65	Yes	A 8-month regimen based on ethambutol and isoniazid	Standard 6-month control	18–20 months	Equivalence [5%]	Completed and published [27]
**NIRT Study 22, CTRI/2012/10/003060**	Reg: 15Oct2012 Trial terminated	India	429	18–70	No, excluded	A 4-month moxifloxacin and ofloxacin regimens given intermittently	Standard 6-month control given thrice weekly	30 months	Equivalence [5%]	Completed and published [28]
**OneRIF,NCT02153528**	Reg: 3Jun2014 PC: 1 Aug2017 SC: 1 Aug2017	Bangladesh	701	≥ 15	No, excluded	High-dose rifampicin regimen for 6 months	Standard 6-month control	18 months	Logistic regression	Completed and published [29]
**STAND, NCT02342886**	Reg: 21Jan2015 PC: Jan2018 SC: May2018	Georgia, Uganda, Malaysia,Philippines, South Africa, Kenya	284	≥ 18	Yes, with restrictions	Pretomanid, moxifloxacin and pyrazinamide for 4- or 6-months	Standard 6-month control	24 months	Non-inferior [12%]	Completed and published [30]
**TBTC Study 31, NCT02410772**	Reg: 8Apr2015 Est. PC: Apr2020 Est. SC: Dec2020	US, Brazil, China, Haiti, Peru, India, Thailand, Vietnam, Kenya, Malawi, S.Africa, Uganda, Zimbabwe	2516	≥ 12	Staged approach, CD4 ≥ 100	A 4-month rifapentine and moxifloxacin regimens	Standard 6-month control	18 months	Non-inferior [6.6%]	Completed and published [31]
**Rifashort, NCT02581527**	Reg: 21Oct2015 Est. PC: Jan2021 Est. SC: Dec2021	Botswana, Uganda, Guinea, Mexico, Nepal, Peru, Pakistan	Target: 654	18–65	No, excluded	A 4-month regimens with high dose rifampicin	Standard 6-month control	18 months	Non-inferior [8%]	Open torecruitment
**NIAID Predict, NCT02821832**	Reg: 4July2016 Est. PC: 31 Dec2021 Est. SC: 31Dec2022	China, South Africa	Target: 1200	18–75	No, excluded	Biomarker-driven 4- or 6-month RIF-based regimen	Standard 6-month control	18 months	Non-inferior [7%]	In follow-up
**Shortened regimens for DS (Pulmonary) TB, NCT02901288**	Reg: 15Sep2016 Est. PC: Dec2018 Est. SC: Dec2018	China	Target: 3900	18–65	No, excluded	A 4.5-month regimen with levofloxacin	Standard 6-month control	28.5 months	Non-inferior [5%]	Completed
**SimpliciTB, NCT03338621**	Reg: 9Nov2017 PC: 30Apr2020 Est. SC: 22Feb2022	Brazil, Georgia, Russian Federation, Malaysia, Phillipines, South Africa, Tanzania, Uganda	455	≥ 18	Yes, with restrictions	Bedaquiline, pretomanid, moxifloxacin, pyrazinamide for 4 months	Standard 6-month control	24 months	Non-inferior [12%]	In follow-up
**Truncate TB, NCT03474198**	Reg: 22Mar2018 Est. PC: 12Mar2022 Est. SC: 12Mar2022	Indonesia, Thailand, Philippines, Singapore	Target: 900	18–65	Staged approach, CD4 ≥200	A 2–3-month regimens with new and repurposed drugs	Standard 6-month control	96 weeks	Non-inferior [12%]	Open to recruitment
**Tri-Do-Re, NCT04260477**	Est. start: 7Feb2020 Est. PC: Nov2024 Est. SC: Nov2024	Niger [9 supported clinics]	Target: 370	No limit	Yes, with restrictions	High-dose retreatment regimen (6R3H3EZ) including pyridoxine	Standard 6-month control	18 months	Logistic regression	Not yet recruiting
**CLO-FAST, NCT04311502**	Est. Start: 17Mar2020 Est. PC: 18Mar2022	Malawi, South Africa, Zimbabwe, Brazil, Peru, Thailand, India, Haiti	Target: 185	≥ 18	Yes, with restrictions	A 3-month rifapentine/clofazimine-containing regimen with CFX loading dose.	Standard 6-month control	65 weeks	Superiority [Time to 12wk culture conversion]	Not yet recruiting
***DR-TB***										
**Stream I, ISRCTN78372190 NCT02409290**	Reg: 14Oct2010 SC: 31July2018	Ethiopia, Mongolia, South Africa, Vietnam	424	≥ 18	Yes	A 9–11-month injectable regimen	Local SOC^c^	132 weeks	Non-inferior [10%]	Completed and published [32]
**Delamanid, NCT01424670**	Reg: 29Aug2011 PC: 4Jul2016 SC: 4Jul2016	Estonia, Latvia, Lithuania, Moldova, Peru, Philippines, South Africa	511	18–69	Yes, with restrictions	Delamanid (added to OBR)	Placebo (added to OBR)	120 weeks	Time to SCC	Completed and published [33]
**Nix-TB, NCT02333799**	Reg: 7Jan2015 PC: 14Jan2019 Est. SC: 13Jul2020	South Africa	109	≥ 14	Yes, if CD4 ≥50 but with restrictions	Linezolid + bedaquiline + pretomanid for 26 weeks	No control group	30–33 months	% with unfavorable outcome	Completed and published [34]
**NEXT, NCT02454205**	Reg: 27May2015 Est. PC: Dec2020 Est. SC: Dec2020	South Africa	154	≥ 18	Yes	A 6–9-month regimen containing bedaquliine and clofazimine and other anti-TB drugs	Conventional SOC regimen in South Africe	21–27 months	Superiority [70%]	Completed
**TB Practecal, NCT02589782**	Reg: 28Oct2015 Est. PC: Dec 2022 Est. SC: Feb2023	Uzbekistan, South Africa, Belarus	Target: 630	≥ 15	Yes	24 weeks of all-oral regimens of bedaquiline, linezolid and pretomanid with or without clofazimine or moxifloxacin	Local SOC^c^	108 weeks	Non-inferior [12%]	Open to recruitment
**Stream II, ISRCTN18148631NCT02409290**	Reg: 10Feb2016 Est. SC:31Jul2022	Ethiopia, Georgia, India, Moldova, Mongolia, South Africa, Uganda	Target: 1155	≥ 15	Yes, if CD4 ≥50	A 6-month injectable regimen, a 9-month all-oral regimen, both with bedaquiline	9–11 month injectable regimen	132 weeks	Non-inferior [10%]	In follow-up
**endTB, NCT02754765**	Reg: 28Apr2016 Est. PC: Sep2020 Est. SC: Apr2021	Georgia, India, Kazakhstan, Lesotho, Pakistan, Peru, South Africa	Target: 750	≥ 15	Yes, with restrictions	A 6–9-month all-oral regimens	Local SOC^c^	104 weeks	Non-inferior [12%]	Open to recruitment
**ZeNix-TB, NCT03086486**	Reg: 22Mar2017 PC: 14Jan2019 Est. SC: 13Jul2020	Georgia, Moldova, Russian Federation, South Africa	181	≥ 14	Yes, with restrictions	Linezolid (at various doses, durations) + bedaquiline + pretomanid after 26 wks of treatment [4 groups]	No control group	104 weeks	Lower bound of 95% CI >50%	In follow-up
**TB-TRUST, NCT03867136**	Reg:7Mar2019 Est. PC: 1Dec2021 Est. SC: 1Dec2022	China	Target: 354	18–70	No, excluded	PZA sensitivity-guided all oral ultra short regimen	WHO standardized shorter regimen	84 weeks	Non-inferior [10%]	Open to recruitment
**endTB-Q,** **NCT03896685**	Reg: 1Apr2019 Est. PC: Oct2022 Est. SC: Dec2022	India, Kazakhstan, Lesotho, Pakistan, Peru, Vietnam	Target: 324	≥ 15	Yes	A 6–9-month all-oral regimen	Local SOC^c^	104 weeks	Non-inferior [12%]	Open to recruitment

^a^Those registration numbers starting with “NCT”: represent ClinicalTrials.gov; “ISRCTN” = International Standard Randomised Controlled Trial Number, and “CTRI” = Clinical Trials Registry of India

^b^“Reg date” The date the trial was first posted on the specified registry. “PC” Primary completion date, the date data collection was completed for all primary outcome measures; “SC” Study completion date, the date the last participant was examined or received an intervention to collect final data for the primary outcomes, secondary outcome measures, and adverse events.”Est.” indicates that date is an estimation

^c^Local standard of care (SOC) consistent with appropriate WHO guidelines at the time

Abbreviations: *SOC* standard of care, *OBR* optimzed background regimen

Ten of the trials targeted patients with drug-resistant TB (DR-TB) and the remaining 21 trials enrolled patients whose TB was drug-susceptible (DS-TB). Two protocols included patients diagnosed with either DS- or DR-TB, although in each case those with DR-TB were enrolled as a non-randomized interventional cohort that was not ”statistically analyzed.” Five (17%) of the trials included participants enrolled in African sites, eight (26%) included participants enrolled in Asian sites, and 16 (52%) included participants enrolled on both continents. Seven (24%) included subjects in South American sites, two (7%) in Latin America and five (17%) in North America. Proposed subjects were as young as 12 (one trial), 14 (two trials), and 15 (four trials), although one trial did not impose a lower age limit; however, most trials included patients aged 18 years and older. Two protocols capped the age of participants at 60, five at 65, one at 70, and another at age 75; in the remaining trials, an upper age limit was not specified. Only one trial exclusively conducted in children and adolescents was included in the 51 trials for protocol and SAP review, but the protocol was not made available for inclusion in our review.

The primary objective uniformly across all but one study was to investigate whether a novel treatment regimen had non-inferior or superior efficacy in terms of a “long-term durable cure extending through post-treatment follow-up.” In the remaining study, efficacy outcomes were secondary to safety outcomes. Novel interventions varied across trials, and included shortening treatment, evaluating the efficacy of new combination regimens, utilizing oral medications exclusively, testing different doses and durations of treatment, testing fixed-dose combination formulations, and simplifying treatment by utilizing intermittent dosing. A non-inferiority analysis comparing a new treatment regimen to standard treatment was specified in 18 (58%) protocols, with margins of non-inferiority ranging from 4 to 12% (median [IQR] 6.6 [5, 8]) for DS-TB trials and 10 to 12% (11 [10, 12]) for DR-TB trials. All margins specified were based on comparisons of composite outcomes. Other techniques used included equivalence testing (n=6), superiority testing (n=3), and logistic regression to compare differences in proportions of participants achieving a favorable outcome (or, conversely, an unfavorable outcome). In 15 (48%) protocols, an intention-to-treat (ITT) or modified intention-to-treat (mITT) analysis was defined as primary, while per protocol (PP) analyses were also planned as secondary or confirmatory analyses. In 14 (48%) studies, the mITT and PP analyses were considered co-primary. In only one of the protocols we reviewed was the PP analysis considered primary; in one other, no specification was made (although in this case we did not have access to the trial SAP).

The duration of experimental treatment regimens ranged from 13 weeks to 26 weeks for DS-TB trials and from 24 to 44 weeks for DR-TB trials. Duration of post-treatment follow-up was of varying lengths; these might be measured as time post-randomization or post-treatment, sometimes in weeks, at others in months. Some protocols specified “time windows” around evaluation dates, while others cited only the week or month representing the end of follow-up without explanation as to how much time before or after defined the follow-up “window.” The total trial duration time from randomization to end of follow-up ranged from 78 to 130 weeks for DS-TB trials and from 104 to 132 weeks for DR-TB trials. In general, the primary trial outcomes were measured at the end of follow-up. At the time of writing, 8 (26%) trials were still open to enrollment. Seven trials (23%) were complete with study findings not yet available or in follow-up, and 2 (6%) were completed and had results posted on ClinicalTrials.gov. For 14 (45%) trials, the primary results of the trial had been published in a peer-reviewed journal or presented at an international conference.

### Outcome definitions

Outcomes across study protocols were assigned to one of three broad categories: favorable, unfavorable, or not assessable. Protocols generally defined an outcome as favorable in terms of timing of culture conversion and required number of negative cultures at the end of the follow-up period. Similarly, determination of an outcome as unfavorable primarily involved the observation of a specific number of positive cultures with or without reference to a time frame for the samples. All protocols specified these bacteriological conditions to some degree, although the circumstances under which determinations were made, and the granularity with which these were defined in individual protocols, varied considerably (see Supplemental Table 1 for a listing of outcome definitions found in protocols). Protocols from recent studies were more likely to allow for categorization of an outcome as not assessable if it could not be clearly classified as favorable or unfavorable, e.g., deaths unrelated to TB, recurrence due to re-infection with a different strain, and loss-to-follow-up with last culture negative. However, in some cases, identical outcome-determining events were categorized as not assessable in some cases and unfavorable in others. For example, while re-infection with a new strain was generally viewed as not assessable, several protocols considered this an unfavorable outcome, and non-TB deaths were almost equally divided between the two categories. Protocols from earlier trials seldom specifically labeled an outcome not assessable, although this designation sometimes could be inferred from descriptions of patients excluded from analyses. In others, however, this possibility was neither explicitly nor implicitly addressed. Outcomes determined to be not assessable will be discussed simultaneously with unfavorable outcomes, since the same event could be interpreted as one or the other by different trial protocols. Table 2 summarizes the range of outcome definitions and the frequency of their occurrence across protocols.

**Table 2:** Summary of reported outcomes, out of 31 clinical trials

Trial event		Outcome timing		Outcome timing	
		During/at the end of treatment [# of studies reporting]	At the end of follow-up [# of studies reporting]	During/at the end of treatment [# of studies reporting]	At the end of follow-up [# of studies reporting]
**Favorable outcomes**					
	Start with negative culture		2		
	Defined by one negative culture: “culture negative” at specified time	2	12		
	Culture(-) not having been retreated or had tx changed or extended		2		
	Defined by two negative cultures, with varying intervals between cultures	1	12		
	Defined by three negative cultures	2	2		
	Defined by all negative cultures in specified time period		1		
	Inability to produce sputum addressed, with various qualifications		10		
	No signs/symptoms and can’t produce sputum		2		
	No signs/symptoms and sputum is contaminated in 2 cultures with no evidence of TB		2		
	Incomplete culture results but clinical symptoms favorable		2		
	Relapse-free cure		2		
		**Unfavorable outcomes**		**Outcome not assessable**	
**Biologically defined**					
	Never convert to culture negative	2	1		
	Do not have negative status, inconsistent qualifications		6		
	Persistently positive after specified time		1		
	Culture positive, confirmed by second (+) sample ≥ 4hr after first (+) culture		2		
	Two or more (+) cultures at least 1 month apart after 2mos in study and still on tx	2			
	Culture positive at end	4	2		
	≥ 1 of last two cultures on separate occasions are (+)		1		
	Culture of ≥20 colonies	1			
	≥ 2 (+) cultures in last month, one of which is 20 colonies or more	2			
**Clinical failure at end, regardless of culture**		2	5		
**Recurrence: Relapse, varying qualifications**		2	29		
**Recurrence: Reinfection with a different strain**			2	3	13
**Death**					
	Any cause	5	7	2	2
	TB-related	11	14		
	Death from extra-pulmonary TB	1			
	Any non-TB-related death	1	1	4	3
	Non-TB, except accident, violence, trauma	7	7	9	1
	Non-TB, except suicide	6	6	3	
	Death from a different TB strain				1
	Died with last culture negative				5
**Withdrawn and lost to follow-up**					
	Did not reach endpoint, varying qualifications		13		
	No culture result at end of FU or last culture contaminated				2
	Culture (+) when last seen	4	4		
	Any withdrawal of or loss to follow-up	2	4	2	2
**Left study with last culture negative**				2	7
**Treatment issues**					
	Extension, with varying exceptions	11	2		
	Restart, with varying exceptions		11		
	Change treatment, with varying exceptions	10	2		
	Initiation of treatment for relapse in absence of culture confirmation		1		
	Change one drug, with varying exceptions	5	3		
	Change more than one drug	5	4		
	Discontinue treatment, with exceptions	10			9
	Discontinue treatment, due to pregnancy			2	
	Incomplete, with varying qualifications	5			
	Off-protocol drugs	3	3		1

Protocols additionally addressed issues around treatment and adherence with respect to categorization of outcome. These will be considered last, as they often coincide with or contribute to other reasons of categorization of outcomes as either unfavorable or not assessable.

### Favorable outcomes

In contrast to unfavorable and not assessable outcomes, favorable outcomes received the most consistent treatment across protocols. In all protocols that we reviewed, a patient with a favorable outcome was defined as one who tested negative on a varying number of cultures, with reference to the end of treatment and/or follow-up. Nonetheless, this seemingly straightforward outcome underwent a multitude of permutations across trials. Some trials required only that a patient be “culture negative;” others defined an outcome as favorable based on a single-negative culture. The majority of trials required at least two negative cultures, and in a small number of trials, a patient was required to have three negative cultures to achieve negative status. In addition to the variability in number of negative cultures required, favorable status was conditional on a variety of restrictions in terms of timing (with reference to either the end of treatment, the end of follow-up or both), spacing (amount of time between the negative cultures that ranged from occurrence on different days to requiring at least four intervening weeks between negative cultures), and culture medium type (solid or liquid).

Spontaneous sputum production usually decreases or resolves during successful treatment and follow-up for TB and most such patients are culture-negative for *M.tb* [35]. A smaller number of studies addressed a patient’s potential inability to produce sputum at various points in the trial as indicative of a favorable outcome. One protocol interpreted a patient’s inability to ever produce sputum as a favorable outcome; another further stipulated that never producing sputum would be considered favorable even if the patient never achieved culture negative status but completed follow-up without clinical or microbiological relapse. Others defined circumstances under which failure to produce sputum at the end of the follow-up period could be classified as negative, e.g., provided this coincided with a patient having prior culture negative status or lacking clinical symptoms. In only one trial was a failure to produce sputum at the end of follow-up categorized as an unfavorable outcome. While generally classified as a not assessable outcome (see below), one study classified patients who developed an infection with a strain different from that with which they had originally been infected (an exogenous reinfection) as having a favorable outcome if the original strain was shown to have been cured. In another, a contaminated culture result or one which could not be evaluated was categorized as favorable, provided there were no positive cultures at the end of follow-up. Two studies allowed for a patient to have had a favorable outcome even with a culture at the end of follow-up that was inconclusive, if clinical and radiological symptoms were supportive of the assessment.

### Unfavorable outcomes vs. not assessable outcomes

In the broadest sense, we found that all the reviewed protocols deemed that a patient’s outcome would be considered unfavorable primarily based on positive sputum cultures. However, the level of detail attached to culture positivity varied from the most general (“failure at end of treatment”) to the bewilderingly complex: in one trial, for example, the outcome of a patient not attending the final visit could not be categorized as unfavorable until all of four specified conditions were met, and two additional conditions had been taken into account.

### Categories of unfavorable/not assessable outcomes

#### Failure to ever achieve negative culture conversion

A patient’s failure to respond successfully, as defined bacteriologically, to the prescribed regimen by the end of the treatment period constituted the most straightforward type of unfavorable outcome. In some protocols, however, the treatment duration could be extended if necessary or if some limited number of treatments had been missed, thus lengthening the time a patient was given to achieve culture conversion or culture negative status.

#### Relapse and re-infection

Recurrence of bacterial infection can occur as an endogenous relapse, defined as a patient’s recurrence with positive culture status with the originally diagnosed strain, having previously attained negative status, or as an exogenous re-infection, i.e., a new infection with a different strain. Not all protocols specifically addressed an analytical approach to both. One protocol did not address either relapse or reinfection; some categorized the status of relapse but not re-infection, and several addressed re-infection but not relapse. Other protocols addressed and categorized both.

#### Relapse

In all studies, relapse was considered an unfavorable outcome in terms of its analytical treatment. Although some studies provided specific definitions of relapse, others included it as either part of a composite outcome or (in a few cases where patients were required to have been previously treated and cured prior to the study) as the primary outcome. Definitions, when provided, varied as to when and how relapse was defined, and with what level of detail, however. Some studies defined a relapse as occurring in patients who were culture-negative at the end of treatment, but with different constraints on the conversion to culture-positive. These included diagnosing relapse in a patient who tested positive twice with no intervening negatives, whose two positive tests occurred at least 1 day apart, who had positive sputum cultures during four consecutive monthly exams (at least one with 20 or more colonies), or who had a subsequent diagnosis and treatment for the same or another DR strain (in a study targeting DR infections). Similarly, two additional DR studies defined relapse as having occurred when a patient was prescribed a new DR regimen after treatment and before the end of follow-up. Another study specified that a patient’s conversion to negative status had to occur over at least 4 weeks, with subsequent positive status (on solid medium) confirmed by a second positive culture on a different day. Other studies offered less specific criteria, including simply “recurrence by the end of the study,” “after cure, single culture positive,” and “one culture positive and clinical features suggestive of recurrent disease.”

#### Reinfection

Unlike relapse, patients who acquired an infection with a different type of TB were regarded by most DR-TB and DS-TB studies as having outcomes that were not assessable. Only one protocol viewed re-infection with a different strain as unfavorable; one study targeting patients with DR-TB categorized re-infection with a *different* DR strain as unfavorable, but with a DS strain as not assessable. As previously mentioned, one protocol categorized a patient’s re-infection as favorable if occurring after a confirmed conversion to negative status with respect to the original strain.

#### Death

With varying degrees of granularity, most protocols addressed death as an outcome, whether occurring during treatment, after treatment during the follow-up period, or during either. One protocol did not mention death in relation to outcome, and another mentioned death only in that it precluded a favorable outcome; we were unable to obtain SAPs for either of these studies. The death of a patient was generally categorized as an unfavorable outcome, although under certain specified circumstances, deaths could also result in study outcomes being considered not assessable.

#### Death during treatment

A patient’s death during treatment could fall into one of the following categories: (1) death due to any cause, (2) death directly related to TB, and (3) death due to causes unrelated to TB. Non-TB deaths were categorized differently across studies; some considered these to be not assessable, while more frequently, studies treated them as unfavorable, with the exception of deaths due to accident, violence, trauma, or suicide (with the exception of suicide, these latter were generally classified as not assessable). Death by suicide was specifically addressed in a third of the protocols, but was considered by some as unfavorable, and by others as not assessable. An additional protocol specified that the outcome of a patient whose death during treatment was unrelated to TB, but whose culture status at the time of death was unknown, would be classified as not assessable.

#### Death during post-treatment follow-up

During the post-treatment follow-up phase, “all cause deaths” (without further differentiation) were regarded as unfavorable outcomes in some studies, while in others, deaths were only considered unfavorable if TB-related. A small number of studies considered a generalized category of non-TB deaths to be not assessable for purposes of analysis. In several studies, the treatment of death during follow-up was determined with respect to bacteriological status. Several trial protocols classified the outcomes of patients who died with their last culture negative as not assessable. Additional studies more specifically proposed that deaths be considered not assessable only if a patient died while culture negative, under the condition that the last positive culture had been followed by two negative cultures at least 7 days apart. In addition, one study specified that a patient who died from extrapulmonary TB would be considered as having an unfavorable outcome; another classified patients whose deaths were due to an infection other than with the originally diagnosed strain to have outcomes that were not assessable.

#### Withdrawal of consent/lost-to-follow up

Across study protocols, outcomes of patients who were lost to follow-up or who withdrew from the study appeared to be the most challenging to categorize. These patients were variously noted as having been lost to follow-up or withdrawn: (1) while still being treated; (2) at any point, during follow-up; (3) after being cured at the end of treatment, during follow-up; or (4) “when last seen.”

#### During the treatment phase

With respect to patients lost or withdrawn while treatment was still ongoing (without further caveats), a quarter of the protocols classified their outcomes as unfavorable; one study alone categorized them as not assessable. Other protocols determined categorization based on the reason for the patient’s withdrawal. In one protocol, patients who withdrew or were lost due to clinical reasons were considered to have an unfavorable outcome. More frequently, patients who exited the study during the treatment phase were considered to have outcomes that were not assessable, including those whose withdrawal was either unrelated to TB or was due to protocol violation, pregnancy, or moving away and/or becoming untraceable at any point.

#### After treatment completion

In addressing patients who were lost to follow-up or who withdrew after completing treatment, unfavorable outcomes could include those who exited the study under any circumstances (although one protocol classified such a patient as having an outcome that was not assessable); those whose last positive culture was not followed by at least two negative cultures ≥7 days apart; those who terminated the study early, but were known to be alive at last contact, or who were lost to follow-up with vital status unknown; patients who had not achieved culture negative status or who had been classified as having an unfavorable outcome before their withdrawal; patients who could not be contacted for some specified period of time prior to the last study visit; and those who had no culture results within a specified window of time prior to the study endpoint. As specified by two protocols, it was also necessary for these latter patients to be either culture positive when last tested, have no other post-baseline results, or have a negative culture at their most recent result, but with radiological or clinical symptoms that were inconclusive.

Alternatively, the following patients were categorized with varying frequency as having outcomes that were not assessable: those whose last culture before study exit was negative; patients whose last two culture results prior to exit were negative, who had not otherwise been deemed unfavorable; patients whose last culture was negative and whose last positive culture was followed by at least two negative cultures at different visits ≥7 days apart, without an intervening positive culture; and patients not otherwise classified as unfavorable prior to exit from study.

Patients who withdrew or were lost to follow-up after having been cured at the end of treatment were specifically addressed by one study; those who either did so with their most recent culture positive or who moved away with their most recent culture positive were considered to have unfavorable outcomes, while those who under the same circumstances were culture negative or whose most recent culture was contaminated were categorized as not assessable.

In some protocols, outcomes were defined at the time when patients “were last seen.” Detailed events included being culture positive, being culture positive with the same type (whether confirmed or not), culture positive not followed by two negatives, or simply not having achieved or maintained culture negative status at the time of their last visit (prior to study endpoint).

### Treatment-related issues (including treatment changes for adverse events)

Most protocols addressed to some extent their analysis plans regarding treatment issues, including extension, restart, change, and discontinuation of the medications which comprised the specific study regimens. Although in most cases patients who experienced treatment disruptions were considered to have unfavorable outcomes, details varied considerably from study to study. A patient whose treatment was extended for any reason was considered to have had an unfavorable outcome by one study. More commonly, however, the outcomes of patients whose treatment was extended were considered unfavorable but with exceptions that were considered not assessable, including temporary drug re-challenge, over-treatment with assigned drugs, ≤21 days non-study anti-TB meds for active TB, secondary isoniazid preventive therapy in HIV+ patients, re-infection, pregnancy, making up missed doses, and remaining on treatment at the end of the study without having been declared a treatment failure.

Some protocols categorized patients whose treatment had to be restarted as experiencing an unfavorable outcome, again with the exceptions that they either had been infected with a different TB type in some cases or had become pregnant in others; another protocol limited designation of an unfavorable outcome to the period after completion of treatment but before the study’s end.

A change in treatment can take many forms, and this was reflected across protocols. A patient who had any change of medication frequency or dose (except in the case of re-infection) was usually considered as having an unfavorable outcome, although two protocols made exceptions for patients with a single drug replacement, or those whose drug replacement was due to a guideline change in the standard of care group (neither affected outcome classification). Patients whose treatment was changed due to clinical or radiological deterioration, or because of non-response or poor adherence, were considered to have an unfavorable outcome by one study each, respectively. Several studies considered as unfavorable outcomes those of patients for whom one drug was replaced or added, while other studies required that a patient’s treatment involve the replacement or addition of at least two drugs. Such categorization based on number of drug changes ranged from the simple to the overly complex: one study placed further conditions on a two-drug change, declaring that it defined a patient’s outcome as unfavorable if this occurred because the patient (1) had not converted by the end of the first (more intense) phase of treatment, (2) had bacteriologically reverted during the second treatment phase after having converted to negative in the first, (3) had evidence of additional acquired resistance to fluoroquinolones or 2nd line injectables, or (4) had not converted their sputum cultures to negative status and had two positive cultures during a specific time period, with the caveat that if one or more of the samples were unavailable or contaminated this would be considered culture positive if the patient displayed deteriorating clinical symptoms.

A patient whose treatment was discontinued was considered by various protocols as having an unfavorable outcome if study treatment was halted for reasons including the following: experiencing a serious adverse event, starting a different DR-TB regimen, and failing to convert after the first phase of a trial where the treatment regimen occurred in two phases, or because the trial regimen needed to be significantly modified for some (unspecified) reason. In most trials, study treatment was discontinued in patients who became pregnant during therapy, who were then treated with standard therapy. In some trials, patients who discontinued treatment because they became pregnant were considered to have an outcome that was not assessable, while in others a patient’s outcome was considered not assessable if the patient’s last culture was negative, but unfavorable if it were positive.

Incomplete treatment in patients whose culture status could not be evaluated at the end of follow-up was considered unfavorable in several studies; an additional protocol defined a patient’s outcome as unfavorable if, in addition to incomplete treatment, a patient had not attained culture negative status by the end of follow-up. The effect of a patient’s missing drugs during the treatment phase was addressed by one protocol that considered this to be unfavorable if some or all drugs were missed regularly, or if all drugs were missed for more than two consecutive weeks.

Patients who took TB-related but off-protocol drugs, or who started TB treatment outside of the study with the most recent culture positive, were considered by one study to have an unfavorable outcome, while off-protocol drugs not related to TB rendered the outcome not assessable. In two other studies, only patients taking specific off-protocol drugs were categorized as having unfavorable outcomes.

## Discussion

In our review of primary efficacy outcomes as defined in the protocols (and SAPs, if available), in 31 confirmatory clinical TB trials for the treatment of active TB, we found broad *conceptual* agreement. A patient’s outcome was classified as favorable or unfavorable based on the number and timing of negative/positive cultures, and most protocols explicitly acknowledged that outcomes were not assessable under certain circumstances (in other cases, this was implicit in descriptions of inclusions and exclusions from given analyses). However, even though they achieved compliance with decidedly broad guidelines for trial sponsors provided by stringent regulatory authorities [36, 37], we found a considerable degree of heterogeneity in outcome definition across trials. In addition, outcomes were for the most part comprised of composite events, and inconsistencies abounded with respect to the ways in which outcome definition was determined by such factors as deviation from treatment regimens, patient withdrawal/loss to follow-up, relapse or reinfection, and even death; the contributions of the individual components to the composite outcome were in all cases unweighted. These outcomes then dictated inclusions and exclusions from different target populations for analysis—ITT, mITT, and per protocol (PP)—which in turn were variously considered to be of primary, secondary, or equal importance; sensitivity analyses were also sometimes variously used.

Nonetheless, we found that certain areas were treated consistently across protocols, indicating implicit areas of consensus that would facilitate standardization of endpoint definitions. The fairly straightforward criteria for determining a favorable outcome allowed this diagnosis to be more easily reached, as compared to an unfavorable or not assessable one. While details differed in terms of the number and timing of cultures indicating conversion, and the duration required to validly declare it a durable, long-term cure, it is probable that a consensus definition of a patient with a favorable outcome would neither be difficult to reach, nor particularly controversial, across investigators and trials. Similarly, a patient who suffered a relapse with the originally diagnosed infecting strain of *M.tb* after reaching confirmed culture negative status was universally classified as having had an unfavorable outcome, although here, too, small variations in definition are needed to standardize this event across trials.

Understandably, standardizing unfavorable outcomes presents a far greater challenge. In a systematic review of outcomes reported in 248 peer-reviewed and published phase III TB studies, Bonnett et al. found substantial differences in the way unfavorable outcomes were defined and implemented across numerous dimensions [38]. That review was limited to data derived from trial publications and included TB trials from 1950 to 2017 (only 18% of which occurred after 1995), yet Bonnett reported inconsistencies as to what constituted an unfavorable outcome even in the most recent trials. In our review, with the granular data obtained from the more necessarily detailed study protocols and SAPs (all of which had been registered since 1995), we likewise found little consensus in the specific details attached to endpoint definitions for unfavorable.

As a result, combining data, interpreting and comparing results, and performing individual patient data (IPD) meta-analyses across trials *that essentially are all working towards the same goal* is at best highly challenging (as experienced with the largest such analysis of TB clinical trial data [4]) and at worst, impossible. The concept of a “favorable” outcome can be more difficult to define for patients who fail to produce sputum, do not complete the trial, or require treatment changes. Distinguishing between unfavorable and not assessable outcomes, as we have shown, presents even greater potential for discordance, and more differing opinions about what event constitutes each. Adding to the confusion is the fact that some patients inevitably exit the study prematurely, and their reasons for not completing the study, along with their culture status at the time of their exit, are inconsistently used to classify their outcomes as unfavorable or not assessable. Even death, an undeniable and immutable outcome, is cause for dissension; while all protocols considered a patient who died from TB to have had an unfavorable outcome, deaths that were not related to TB could be interpreted as unfavorable or not assessable, depending on circumstances. The conventional use of composite outcomes, which may or may not be directly related to treatment, and that involve numerous assumptions about each “piece” of the outcome, further clouds evaluations of efficacy.

The ICH E9 (R1) addendum [14], in providing a framework and language for defining clinical trial estimands and outcomes, directly addresses many of these problems. While no guidelines can cover all circumstances that may arise in a trial, and it is unlikely that one estimand will satisfy the interests of all categories of trial stakeholders, interpretation and comparison across trials would be greatly facilitated by a standardization of the elements of a trial used to evaluate the efficacy of its intervention. In our review of protocols, we found a wide range of granularity of definitions. While some protocols defined outcomes in the most general terms, others complicated definitions by attempting to cover every possible eventuality; in the latter case, the outcome definitions were clouded with minutia, making consensus with other trials extremely unlikely. On the other hand, the absence of precise definitions of outcomes in the protocol or SAP means that some classification decisions are left to the data analyst; these may have only been “documented” in the analysis code, which is rarely reviewed by study investigators. The ICH E9 (R1) addendum has taken an instructive approach in addressing these problems, separating from the definition of the primary outcome, called an estimand, the many events that occur and either preclude or affect observation of an outcome (referred to by the ICH E9 as “intercurrent events”). This allows not only for a consistent definition of the primary efficacy outcome across trials but also gives a structure for specifying how intercurrent events will be handled in the analysis, thus reducing potential inflation of type I and type II errors. Events that have until now been viewed as rendering an outcome “unfavorable” or “not assessable” (see categories of unfavorable/not assessable above) can rather be categorized as intercurrent events, with decisions about how they will be dealt with in analyses made prior to the beginning of the trial, based on the defined estimands. Thus, within the same trial, an intercurrent event may be treated one way for one estimand, and another way for a second estimand, dependent on the needs of particular stakeholders. Bringing such events to the forefront therefore would allow for standardization in the way they are classified and how they are treated in the analysis, resulting in the reporting of outcomes that are comparable across trials. Even if *standardization* is not possible between different trials, at the very least the ICH E9 (R1) addendum provides a lingua franca for *specification* to support clear interpretation and translation into clinical practice guidelines. Some preliminary work has been done exploring the role of the estimand framework in the context of individual TB trials [39, 40].

It is worth recognizing that the estimand framework will not remove all the challenges in choice of analysis populations and choice of appropriate analytic techniques. On the one hand, different estimands can be described to mimic certain analysis populations, thereby retaining some of the same limitations; on the other hand, the estimand framework does not address the actual task of estimation to yield a sufficiently precise and unbiased estimate of a treatment effect. Nevertheless, the clear taxonomy of the estimand framework, if properly applied, better facilitates a discussion as to which estimands are more or less appropriate. It also clearly delineates the description of the treatment effect of interest from the method of estimation, whereas in the past the former is sometimes informed and limited by the latter.

Our study has several limitations. We were not able to obtain an SAP for every trial we reviewed and for which we obtained protocols; for these studies, therefore, we did not have access to the more detailed descriptions of how events would be dealt with in analyses that an SAP provides. The sheer length of the protocols themselves made locating specific pieces of information difficult (and in some cases, particularly in the case of older studies, it was not present or was treated in general terms, with specifics purportedly left to the SAP). Furthermore, even in trials where SAPs were available, the information was sometimes insufficient to fully describe the endpoint—presumably, some decisions on endpoint classification were only documented in trial team meeting minutes or in analysis code written by the study statistician. While our search for clinical trials was as thorough as possible, we cannot be sure that all recent clinical trials were included. We did not receive responses from investigators for 17 of the 51 trials selected for protocol review. These were necessarily excluded, although many may not have been within the scope of our review (notably some of the country-specific trial registries had limited data to assess whether trials were within scope). We did not distinguish trials of unlicensed drugs conducted under stringent regulatory oversight authorities and sometimes sponsored by the pharmaceutical industry from the investigator-initiated trials of licensed drugs. Both are designed to inform policy and practice, and better specification and standardization of endpoint definitions is relevant to all future TB treatment trials.

While no estimand can include all possible trial occurrences, the standardization of definitions and of the treatment of intercurrent events that occur most frequently will enhance comparability across trials, while allowing for interpretation of rare or unanticipated events. The approach outlined by the ICH E9 addendum can also be used to develop different estimands to address the concerns of specific audiences. It is therefore important, following the recommendations of the ICH E9 addendum, to prioritize both the specification and standardization of outcomes across TB trials. As new drugs and treatment regimens are discovered and tested in trials, the ability to make valid comparisons to old treatments and regimens is also essential if researchers are to effectively collaborate towards our common goals of developing shorter, simpler, and more effective and safe treatment to cure patients with TB. Following this review, our next step will be to produce recommendations for estimands and methods of estimation for TB treatment trials. At a time when the world has begun to establish large adaptive platforms with core protocols for the search of active treatment of COVID-19, it is past time that we, as a TB community, move towards better standardization and harmonization of trial methods.

This work was supported, in whole or in part, by the Bill & Melinda Gates Foundation [Grant #INV-002039]. Under the grant conditions of the Foundation, a Creative Commons Attribution 3.0 Generic License has already been assigned to the Author Accepted Manuscript version that might arise from this submission.

## Supplementary Informations

**Additional file 1: Supplemental Table 1.** Listing of outcome definitions across protocols.

## Data Availability

Not applicable
