# Supplementary material for: A systematic review of endpoint definitions in late phase pulmonary tuberculosis therapeutic trials
Source: Trials. 2021 Aug 3;22:515. doi: 10.1186/s13063-021-05388-1 (PMC8329622; doi:10.1186/s13063-021-05388-1)
Supplement: Supplementary file 1 — Additional file 1: Supplemental Table 1. Listing of outcome definitions across protocols. [file 13063_2021_5388_MOESM1_ESM.docx]

| Supplemental Table 1. Listing of outcome definitions across protocols | |
| --- | --- |
| **FAVORABLE OUTCOME** | |
| **Defined in terms of 2 negative cultures** | |
|  | Culture (-) at 6 months from end of treatment and not already classified as having an unfavorable outcome, and last (+) culture result followed by ≥ 2 (-) cultures. |
|  | Culture (-) status at time of endpoint (at 52 or 104 weeks), and not classified as having an unfavorable outcome and last (+) culture result followed by ≥ 2 (-) cultures. |
|  | Negative culture status at 12 months from start of therapy (50-54 weeks) and not classified as having an unfavorable outcome, and last (+) culture followed by ≥ 2 (-) cultures. |
|  | Negative status at 18 months (at or after 72 weeks), not classified as unfavorable, and last (+) culture followed by ≥ 2 (-) cultures. |
|  | Two (-) cultures > 1 day apart at end of treatment (16/24 weeks). |
|  | Last 2 cultures (-) taken on separate visits (on different days); the latest of which is in the Week 132 visit and NO previous classification as unfavorable. |
|  | Last 2 cultures (-) taken on separate visits (on different days); the latest of which is in the Week 76 visit and NO previous classification as unfavorable. |
|  | No culture result within Week 132 window because patient couldn't produce sputum, if last 2 cultures (-) and not previously classified as unfavorable. |
|  | No culture result within Week 76 window because patient couldn't produce sputum, if last 2 cultures (-) and not previously classified as unfavorable. |
|  | At least 2 consecutive (-) cultures on solid medium over a span of at least 4 weeks achieved before end of treatment, with no subsequent confirmed (+) cultures in follow-up. |
|  | Two consecutive (-) cultures on liquid medium over a span of at least 4 weeks for patients who were liquid medium (+) but solid medium (-) at baseline. |
|  | Last 2 cultures (-), taken on separate visits, latest within 9 weeks of end of study. |
|  | At least 2 consecutive (-) sputum cultures taken ≥ 4 weeks apart (±2 weeks) at 72 weeks post-randomization. |
|  | Single (-) culture followed by ≥ 1 confirmatory single (-) culture ≥ 25 days after the first (-) and not followed by a confirmed (+), ≥ 2 (+) single culture results, not counting intermittent, missing, or contaminated results in 30 months follow-up and being alive at the last contact for follow-up. |
| **Defined in terms of 3 negative cultures** | |
|  | Treatment completed as per regimen without evidence of failed treatment (or other unfavorable outcome) and with ≥ 3 consecutive cultures (-) taken ≥ 30 days apart by month 7 or earlier. |
|  | Treatment completed as recommended without evidence of failed treatment (or other unfavorable outcome) and ≥ 3 consecutive cultures taken ≥ 30 days apart are (-) after intensive phase. |
|  | At end of treatment, all three cultures are negative in last month. |
| **Defined in terms of negative cultures, but "culture negative" not specifically defined** | |
|  | Culture (-) at end of treatment and maintained until follow-up at 18 mos. |
|  | Culture (-) at end of treatment and culture (-) ≥ 1mo later without TB event between end of treatment and end of long-term follow-up. |
|  | After cure, culture (-) and data available for all visits or data available for last FU visit. |
|  | Culture (-) at end of treatment and patient is lost to follow-up. |
|  | Culture (-) at end of treatment and patient died (from death not related to TB). |
|  | Culture (-) at the end of scheduled follow-up, not otherwise classified as having an unfavorable status. |
| **Patient can't product sputum** | |
|  | Patient never achieves culture (-) status due to inability to produce sputum, after TB confirmation via culture has been confirmed on the applicable baseline sample but completes 12/24 months follow-up without clinical or microbiological evidence of relapse. |
|  | Unable to produce sputum. |
|  | Unable to produce sputum at 72 weeks but had culture (-) status earlier. |
|  | All sputum cultures negative during the last 2 months of treatment and completed 12 months of follow-up without recurrence. |
|  | At 18 months or later, clinically asymptomatic and unable to produce sputum and not already classified as having an unfavorable outcome. |
|  | No culture result within Week 132 window because unable to produce sputum, if last 2 cultures (-) and not previously classified as unfavorable. |
|  | No culture result within Week 76 window because unable to produce sputum, if last 2 cultures (-) and not previously classified as unfavorable. |
| **Clinically favorable** | |
|  | "Favorable outcome" (undefined) at 72 weeks post-randomization. |
|  | Discordant cultures found (at a time defined by the method used and the timing of clinic visits), and a third culture is taken and is (-). |
|  | Last culture result (within 9 weeks of end) is (-) and no other post-baseline culture result and favorable bacteriological/radiological/clinical evolution. |
|  | Last culture result (within 9 weeks of end) is (-) and penultimate (+) due to cross-contamination and favorable bacteriological/radiological/clinical evolution. |
|  | No culture in 9 weeks prior to end of study and most recent culture is (-) and favorable bacteriological/radiological/clinical evolution. |
|  | Culture in 9 weeks prior to end (+) due to cross-contamination (cc) and most recent culture is (-) and favorable bacteriological/radiological/clinical evolution. |
|  | At 18 months or later, clinically asymptomatic and unable to produce sputum and not already classified as having an unfavorable outcome. |
|  | Patient who at week 96 does not still require TB treatment, does not have ongoing TB disease activity, and has not died (unless death is unrelated to TB). |
| **Miscellaneous** | |
|  | After cure, 2 cultures (+) at least 1 day apart and organism is NOT *mTB.* |
|  | Missing data of LJ culture at 72 weeks and MGIT negative. |
| **UNFAVORABLE OUTCOME** | |
| **Bacteriological failure** | |
|  | Patient is not culture negative status at time of endpoint. |
|  | Failure at end of treatment. |
|  | Failure to achieve sputum culture conversion by end of follow-up. |
|  | Last positive culture not followed by at least 2 (-) results. |
|  | At least 1 of last 2 cultures (one within 9 weeks of endpoint) is (+) in the absence of evidence of cross-contamination. |
|  | Last culture (-) between Weeks 65-73 and no other post-baseline result and bacteriological/radiological/clinical evolution unfavorable. |
|  | Last culture (-) between Weeks 65-73 and penultimate culture (+) due to cross contamination and bacteriological/radiological/clinical evolution unfavorable. |
|  | No culture result within 9 weeks of endpoint and most recent culture (-) and bacteriological/radiological/clinical evolution unfavorable. |
|  | Culture result within 9 weeks of endpoint (+) due to cross-contamination and most recent culture (+) in absence of cross contamination. |
|  | No culture result within 9 weeks of endpoint and most recent culture (+) in absence of cross contamination. |
|  | Culture result within 9 weeks of endpoint (+) due to cross contamination AND most recent culture (-) and bacteriological/radiological/clinical evolution unfavorable. |
|  | No culture result within 9 weeks of endpoint and no other post-baseline culture result. |
|  | No culture result within 9 weeks of endpoint and most recent culture (+) due to cross contamination. |
|  | No culture result within 9 weeks of endpoint and most recent culture (-) and bacteriological/radiological/clinical evolution not assessable. |
|  | Culture result within 9 weeks of endpoint (+) due to cross contamination and no other post-baseline culture result. |
|  | Culture result within 9 weeks of endpoint (+) due to cross contamination and most recent culture (+) due to cross contamination. |
|  | Culture result within 9 weeks of endpoint (+) due to cross contamination and most recent culture (-) and not assessable. |
|  | "Previously classified as unfavorable" (unless not assessable at Week 39; then eligible for re-evaluation at Week 73). |
|  | One or more cultures (+) in last month of treatment, one of which is at least 20 colonies. |
|  | Two or more (+) during last 2 months of treatment period (months 4-6). |
|  | Two or more (+) during Weeks 65-73. |
|  | Lack of conversion by end of intensive phase. |
|  | Persistent smear (+) or culture (+) after 4th month. |
|  | Remain culture (+) on solid medium at Week 24. |
|  | Convert to solid culture (-) and subsequently have single (+) before or at Week 24 and then have second (+). |
|  | Become solid culture (+), then a second (+), before/at Week 24 after solid culture (-). |
|  | Culture (+) on solid medium between and including Weeks 16-24 confirmed on subsequent culture. |
|  | Previously culture (-) status, patient, after treatment, has 2 (+) cultures w/out an intervening negative. |
|  | Patient culture (+) at end of treatment and with another sputum sampled 1 day or more later with culture (+). |
|  | One or more cultures (+) from specimens taken on separate occasions. |
|  | No culture result at Wk76 window. |
|  | Unfavorable outcome at or before end of treatment. |
|  | Failing to attain culture (-) status and being declared unfavorable. |
|  | Two separate cultures (+) ≥ 4wks apart (± 2wks) from Weeks 28-108. |
|  | Two separate cultures (+) ≥ 4wks apart from Week 16 (± 2 weeks) or later. |
|  | Composite outcome comprising all-cause death, treatment failure or discontinuation, loss to follow-up, still on treatment at end of study (108 weeks) or recurrence. |
|  | Patient acquires resistance. |
|  | Patient acquires resistance to fluoroquinolones or second line injectables. |
|  | Patient receives new MDR-TB regimen after end of treatment and before final endpoint. |
|  | Treatment failure (culture confirmed). |
|  | Treatment failure (not culture confirmed). |
| **Clinical failure** | |
|  | Treatment change due to clinical or radiological deterioration due to TB. |
|  | New clinical or lab worsening if additional meds are needed. |
|  | Patient has surgery and resected tissue cultured and (+). |
|  | Ongoing TB disease activity at week 96 (2 out of A, B, C, or if D): A) clinical evidence, B) CXR evidence, C) microbiological evidence (presumptive)=one sample culture (+) within Week 96 analysis window, D) microbiological evidence (conclusive)=culture (+) on samples taken on separate days with at least one taken in Week 96 window. |
|  | Same as above, for non-pulmonary TB (all from same site). |
|  | Develop extra pulmonary TB. |
|  | Ongoing TB activity at Week 96. |
| **Relapse** | |
|  | Culture (-) at end of treatment, but then become (+) with same strain. |
|  | Culture (-) or doubtful bacteriological response at end of treatment and 2 (+) cultures in a 2-mo period, one of which is at least 20 colonies. |
|  | Culture (-) or doubtful bacteriological response at end of treatment, and positive sputum cultures during four consecutive monthly examinations, one of which is at least 20 colonies. |
|  | Two (+) cultures within 4 months without intervening negative culture. |
|  | Culture (-) at end of treatment, then 2 consecutive (+) cultures at least one day apart during follow-up with same type. |
|  | Patient completed treatment without being declared a failure and subsequently diagnosed and require MDR-TB treatment for same strain. |
|  | Become (+), then second (+) on subsequent culture while on treatment after 2 consecutive (-) over at least 4 wks. |
|  | If clinical features suggest recurrent disease, only one (+) will be required to define relapse. |
|  | Reversion in continuation phase after conversion to negative. |
|  | Become positive after 2 culture (-) during follow-up after Wk24. |
|  | Two (+) cultures in 2 sputum collections taken consecutively ≥ 1 day apart during follow-up period after culture-proven cure at end of treatment. |
|  | After cure, 2 (+) cultures in 2 sputum collections taken consecutively ≥ 1 day apart after end of treatment. |
|  | After cure, one culture (+) and second not done or contaminated. |
|  | Culture (-) status at end of treatment, then 2 (+), no intervening negative. |
|  | After cure, single culture (+) and documented evidence of starting TB treatment in follow-up. |
|  | Recurrence (by 18 months). |
|  | Patient completed treatment without being declared a failure and has subsequently been diagnosed and requires MDR-TB treatment (with evidence that the recurrence is due to an MDR or XDR TB strain). |
|  | Convert to culture (-) over at least 4 weeks and subsequently become (+) on solid medium, during follow-up after Wk24, confirmed by a second (+) on another day. |
| **Reinfection with a different strain** | |
|  | Complete treatment without being declared a failure and subsequently diagnosed and require MDR-TB treatment, but due to a different strain. |
|  | Failure/recurrence specimen at/before Week 76 window with a different strain. |
|  | Failure/recurrence specimen at/before Week 104 window with a different strain. |
| **Death** | |
|  | Death during treatment (due to TB). |
|  | Due to TB and another disease. |
|  | Death from any cause during treatment. |
|  | Death from any cause during follow-up. |
|  | Death with documented evidence not due to TB. |
|  | Non-TB death (accident, trauma, suicide, opportunistic infection). |
|  | Suicide. |
|  | Any death but accident, trauma, or violent cause during treatment. |
|  | Due to TB during follow-up. |
|  | Death from confirmed/suggested possible treatment failure or relapse. |
|  | Patient completed ATT but died due to TB during 12 months of follow-up. |
|  | After cure, death during lost to follow-up and one culture (+) based on most recent culture results. |
|  | After cure, single culture (+) and death (with evidence of TB as cause of death). |
|  | After cure, death during follow-up and one culture (-) based on most recent culture results (or culture contaminated). |
| **OUTCOME NOT ASSESSABLE** | |
| **Reinfection with a different strain** | |
|  | Culture (-) at end of treatment, then 2 (+) consecutive cultures at least one day apart during follow-up with a different strain. |
|  | Re-infected with a different strain after converting to culture (-). |
|  | Re-infected with a different strain. |
|  | Patient completes treatment without being declared a failure and is subsequently diagnosed and requires MDR-TB treatment, but due to a DS strain. |
|  | Failed treatment (during treatment phase) but re-infected with another strain during treatment. |
| **Death** | |
|  | Any cause death. |
|  | Non-TB death (accident, trauma, suicide, opportunistic infection). |
|  | Suicide. |
|  | Death during follow-up with no evidence of failure or relapse of TB. |
|  | Death where patient had a study visit at or after Week 48, was not on treatment and had no evidence of ongoing TB activity when last seen, and where cause of death is known to be unrelated to TB or TB drugs. |
|  | Death during follow-up with no evidence of failure or relapse of TB, last culture (-) and last (+) result followed by at least 2 (-) cultures at different visits (at least 7 days apart) and who has not been classified as unfavorable. |
|  | Death from a TB strain different from the originally diagnosed strain. |
|  | Death with last culture (-). |
| **Lost to follow-up or withdrawn** | |
|  | Any withdrawal or lost to follow-up. |
|  | After cure, lost to follow-up, moved away and most recent culture (+). |
|  | After cure, lost to follow-up, moved away and most recent culture (-) or contaminated. |
|  | After cure, withdrawal of consent and most recent culture (-) or contaminated. |
|  | Lost during follow-up and most recent culture (-) or contaminated. |
|  | Lost to follow-up after end of treatment, with last culture (-). |
|  | Left study with last culture (-). |
|  | No culture result at end of follow-up or last culture contaminated. |
|  | Withdrawn from study because of protocol violation. |
|  | Moved away with documented evidence that move is unrelated to TB. |
|  | After treatment, last status culture (-) and last (+) culture followed by at least 2 (-) culture results at different visits (≥ 7 days apart), without intervening (+) culture. |
| **Treatment related** | |
|  | Discontinue drugs due to pregnancy. |
|  | Discontinue drugs due to pregnancy, last culture (-). |
|  | Modify treatment for other than unfavorable response. |
|  | Start taking prohibited meds, but not TB-related. |
| **Other** | |
|  | Culture result within Week 76, but not within Week 132 window, having not otherwise been classified as unfavorable, and last 2 cultures from specimens taken on different occasions, are negative. |
|  | Able to produce sputum at primary endpoint visit, but sputum samples are all contaminated or missing, can't come back for repeat cultures but hasn't been classified as unfavorable and last (+) culture followed by at least 2 (-) cultures. |
